# In Situ-Forming Cellulose/Albumin-Based Injectable Hydrogels for Localized Antitumor Therapy

**DOI:** 10.3390/polym13234221

**Published:** 2021-12-01

**Authors:** Ying Chen, Xiaomin Wang, Yudong Huang, Peipei Kuang, Yushu Wang, Yong Liu, Weihan Yin, Jiahui Zan, Yupeng Liu, Chao Yin, Quli Fan

**Affiliations:** 1 State Key Laboratory of Organic Electronics and Information Displays & Institute of Advanced Materials (IAM), Nanjing University of Posts & Telecommunications, 9 Wenyuan Road, Nanjing 210023, China; iamyingchen@njupt.edu.cn (Y.C.); hyd19961213@163.com (Y.H.); k904727086@163.com (P.K.); 17705992570@163.com (Y.W.); lyhg_fighting@163.com (Y.L.); yinweihan1998@foxmail.com (W.Y.); 17805050309@163.com (J.Z.); 2 School of Integrative Medicine, Tianjin University of Traditional Chinese Medicine, Tianjin 301617, China; wangxiaomin0914@126.com; 3 Key Laboratory for Biomass Energy and Material, Institute of Chemical Industry of Forest Products, CAF, Nanjing 210042, China; 4 Co-Innovation Center of Efficient Processing and Utilization of Forest Resources, Nanjing Forestry University, Nanjing 210037, China

**Keywords:** injectable hydrogel, cellulose/albumin-based, hydrogen bonding, localized antitumor therapy

## Abstract

Injectable hydrogels, which are formed in situ by changing the external stimuli, have the unique characteristics of easy handling and minimal invasiveness, thus providing the advantage of bypass surgical operation and improving patient compliance. Using external temperature stimuli to realize the sol-to-gel transition when preparing injectable hydrogel is essential since the temperature is stable in vivo and controllable during ex vivo, although the hydrogels obtained possibly have low mechanical strength and stability. In this work, we designed an in situ fast-forming injectable cellulose/albumin-based hydrogel (HPC-*g*-AA/BSA hydrogels) that responded to body temperature and which was a well-stabilized hydrogen-bonding network, effectively solving the problem of poor mechanical properties. The application of localized delivery of chemotherapeutic drugs of HPC-*g*-AA/BSA hydrogels was evaluated. In vitro and in vivo results show that HPC-*g*-AA/BSA hydrogels exhibited higher antitumor efficacy of reducing tumor size and seem ideal for localized antitumor therapy.

## 1. Introduction

In situ-forming injectable hydrogel has attracted wide attention for its unique advantages, such as sol-to-gel transition, shape adaptation, non-invasive implantation, easy encapsulation, high payloads, etc. [1,2,3]. So far, injectable hydrogel has been reported for various biomedical applications, including drug delivery [4], cartilage repair [5], cell encapsulation [6], and tissue engineering [7]. For example, a hydrogel formulation solution was injected into the irregular tissue defect formed after tumor resection to form a gel to fill the deficiency in response to a physiological condition, allowing minimal invasive implantation and providing the benefits of retention in the desired location. Various stimuli lead to in situ gel formation, involving temperature differentials [8], pH shift [9], UV-irradiation [10], and solvent exchange [11], among which the thermo-sensitive approach is fundamental since the temperature is both stable in vivo and controllable during ex vivo. However, limited mechanical strength and low stability are the main debits of thermo-sensitive injectable hydrogels, probably resulting from swelling or dissolution of the polymers. To improve the hydrogel strength and stability, covalent (chemical) or nonvalent (physical) crosslinking strategies are employed to develop cross-linked thermosentitive hydrogels [12,13,14,15].

In addition to mechanical strength and stability problems, biocompatibility is also necessary when designing in situ-forming injectable hydrogels for biomedical applications. Using natural polymers such as polypeptides [8,16,17] and polysaccharides [18,19,20] is an excellent strategy for ensuring biocompatibility in the hydrogels. Injectable hydrogels from natural polymers via the sol-to-gel phase transition route are appealing for biomedical applications because of their biodegradation, bioactivity, and intrinsic similarities to the extracellular matrix of the tissues [21]. Among the natural systems, alginate [22], chitosan [23], collagen [24], hyaluronic acid [25], gelatin [26], dextrin [27], fibrin [28], and silk [7] are the most used. Albumin is an abundant and renewable protein material in nature, widely considered for biomedical applications for its biocompatible, biodegradable, and non-toxic nature. Several albumin-based hydrogels were reported in the literature. For example, Qian et al. prepared an injectable hydrogel-encapsulating paclitaxel-based nanoparticle (PRNP-gel) according to the temperature-responsive phase transition of poly(ethylene glycol)-bovine serum albumin (PEG-BSA) [29]. Bubpamala et al. fabricated a pH-stimulus injectable hydrogel constructed by a crosslinked network, which contained poly(ethylene glycol), tannic acid, bovine serum albumin, and Fe(III) for the use of controlled drug release [30].

Herein, we report the design of an in situ fast-forming injectable hydrogel at body temperature stabilized through a hydrogen-bonding network. The primary components of this hydrogel—hydroxypropyl cellulose (HPC) and BSA—are among the most widely used biocompatible polymers, though BSA might cause an allergic response to some people [31,32]. We chose HPC as the polymer backbone to form hydrogels since it is a thermosensitive polymer with an LCST of about 41 °C and a valuable tool for applications in biological and medical fields due to its bio-compatibility approved by the US Food and Drug Administration (FDA). We coupled the abietic moiety from natural rosin as a hydrophobic moiety with the HPC macromolecular to adjust the LCST of the HPC to fit the body temperature of 37 °C to optimize the HPC at the desired property of temperature-induced gelation. The HPC-*g*-AA/BSA hydrogels were formed by a sol-to-gel transition at body temperature, which could serve as drug depots that transport and sustain the release of therapeutics in a controlled way. The gelation behavior and rheological analysis of the hydrogels was studied. The biocompatibility of the hydrogels was evaluated in vitro/vivo. Moreover, the HPC-*g*-AA/BSA hydrogels were injected into the tumor-bearing mice and assessed the potentiality of the hydrogel for localized antitumor therapy.

## 2. Materials and Methods

### 2.1. Materials

Hydroxypropylcellulose (HPC, molecular weight (Mw) 100,000, Polydispersity Index (PDI) = 1.09), bovine serum albumin (BSA), abietic acid (AA), and 4-toluene sulfonyl chloride (TOS-Cl) were bought from Sigma-Aldrich Co., LLC (St. Louis, MO, USA). Doxorubicin hydrochloride (DOX) was purchased from the Aladdin Chemical Reagents Company. Hydrochloric acid, petroleum ether, dimethylacetamide, and other chemical reagents were used without any further purification.

### 2.2. Cell Lines and Animals

Mouse embryo cells, 3T3 cells, and murine mammary carcinoma cell line 4T1 were purchased from the Shanghai Institute of Cell Biology (Shanghai, China). The male BALB/c mice (aged 6–8 weeks, weighing 16–20 g) were obtained from the Experimental Animal Center of Nanjing Drum Tower Hospital. All animal experiments were performed under a protocol approved by the Ethics Review Board for Animal Studies (IACUC-002-27) of KeyGen BioTECH (Jiangsu, China).

### 2.3. Synthesis of Hydroxypropylcellulose-graft-Abietic Acid (HPC-g-AA)

**Scheme 1:**
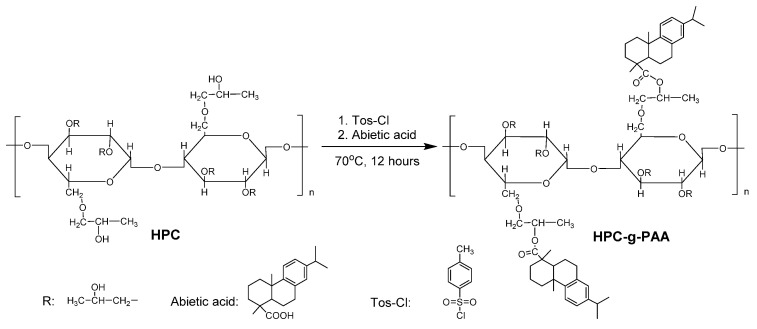
The synthesis route of HPC-*g*-PAA.

We synthesized three HPC-*g*-AA with different degrees of substitute (DS) of AA by editing the molar feed ratio (0.6, 0.8, and 1.0) of the carboxyl group on the AA/hydroxyl group on the HPC unit. Take the HPC-*g*-AA_1.0_ (molar feed ratio = 1.0) as a typical example (Scheme 1): 5.0 g (0.015 mol) of HPC was dissolved in 125 mL of DMAC; then, 2.84 g (49.5 mmol) of Tos-Cl was added to HPC solution and stirred at 70 °C for 3 h; 4.50 g (0.015 mol) of AA was added to the above solution. The mixture reacted at 70 °C under stirring overnight, followed by removing the solvent using vacuum distillation. The products of HPC-*g*-AA were precipitated in 500 mL petroleum ether and washed three times with 250 mL petroleum ether. The average DS could be calculated by comparing the relative intensities of the AA and HPC proton, as described by the following equation:

*DS* = 8(I_peak at 7.7–7.8 ppm_ + I_peak at 7.2–7.3 ppm_)/3(I_peak at 3.2–3.60 ppm_ + I_peak at 3.60–4.00 ppm_) (1)

### 2.4. ^1^H NMR Characterization

^1^H NMR (300 MHz) spectra were recorded on Bruker AVANCE III spectrometer at room temperature with CDCl_3_ as the solvent.

### 2.5. Preparation of HPC-g-AA/BSA Hydrogels

Bovine serum albumin precursor solutions (BSA, 10 wt%) were prepared, and the the pH of the BSA solution was adjusted to pH = 4.0 using 1 M HCl at room temperature. After mixing with an aqueous solution of HPC-*g*-AA (10 wt%), HPC-*g*-AA/BSA hydrogels were formed at 37 °C in a few minutes. Solutions were allowed to gel completely for 2 h.

### 2.6. Rheological Analysis

Rheological measurements were carried out on a DHR-I rheometer (TA Instruments, Italy) equipped with parallel-plate geometry (diameter = 40 mm). Oscillatory experiments (time, frequency, and temperature sweeps) were performed on hydrogels to evaluate their injectability and stability. The parameters set up for each experiment are indicated in Table 1.

### 2.7. Preparation of DOX@HPC-g-AA/BSA Hydrogels

Bovine serum albumin precursor solutions (BSA, 10 wt%) were prepared. Then the pH of the BSA solution was adjusted to be 4.0 using 1 M HCl at room temperature. An amount of DOX was dissolved in the BSA solution to obtain the desired concentration. After mixing with an aqueous solution of HPC-*g*-AA (10 wt%), DOX@HPC-*g*-AA/BSA hydrogels were formed at 37 °C for 10 min.

### 2.8. Biocompatibility Studies of HPC-g-AA/BSA Hydrogels

MTT assays were carried out to assess the biocompatibility of the HPC-*g*-AA/BSA hydrogels in vitro. An amount of 3T3 cells (5000 cells/well) were cultured in DMEM medium containing 10% FBS and 1% penicillin/streptomycin at 37 °C under 5% CO_2_ for 24 h, and then, a different desired amount of HPC-*g*-AA/BSA hydrogels (final concentration per well: 0.032, 0.063, 0.25, 0.5, 1, 2 g/L, respectively) was added into the cultured cells. After incubation for 24 h, MTT assays were used to determine cell viability. All experiments were performed five times, and all the data are presented as the averaged results and standard deviation.

### 2.9. Cytotoxicity of DOX@HPC-g-AA/BSA Hydrogels

MTT assays were used to assess the cell viability of 4T1 cells after incubation with the DOX@HPC-*g*-AA/BSA hydrogels. The 4T1 cells (5000 cells/well) were cultured in DMEM medium containing 10% FBS and 1% penicillin/streptomycin at 37 °C under 5% CO_2_ for 24 h, and then DOX@HPC-*g*-AA/BSA hydrogels and DOX (final DOX concentration per well 0.1, 0.2, 1.0 mg/mL for both samples) were added into the cultured cells separately. After incubation for 24 h, MTT assays were used to determine cell viability. All experiments were performed three times, and all the data are presented as the averaged results and standard deviation.

### 2.10. In Vivo Antitumor Efficacy

The 4T1 cells suspended in a 50% *v*/*v* mixture of Matrigel in supplemented DMEM were subcutaneously injected into the left axilla of the BALB/c male mouse (20 g) to establish a tumor model. Mice bearing tumors were randomly grouped into five groups (n = 4). An amount of 5% saline, free DOX, HPC-*g*-AA/BSA hydrogels, and DOX@HPC-*g*-AA/BSA hydrogels with an equivalent DOX dose of 2 mg kg^−1^ was administered through tail vein intravenous or subcutaneous injection. The antitumor efficacy was evaluated by tumor volumes, calculated by the following equations: Tumor volume (mm^3^) = 0.5 × width*width^2^. The body weight was recorded as well every two days.

### 2.11. Histological Studies

The mice were sacrificed after 16 days of treatments. For all groups, the tumors of mice were collected and fixed in 4% formalin. The tumor tissues were then cut into sections for proliferating cell nuclear antigen (PCNA) and terminal deoxyribonucleotidyl transferse (TdT)-mediated biotin-16-dUTP nick-end labeling (TUNEL) staining based on the standard protocol. The obtained slices were captured under a laser scanning confocal microscope (LCSM). For DOX@HPC-*g*-AA/BSA hydrogel-treated mice, the heart, liver, spleen, lung, kidney, and intestine were harvested and fixed in 4% formalin. Paraffin-embedded sectioning was then performed for hematoxylin and eosin (H&E) staining according to standard protocol, and the images were captured using a digital microscope.

## 3. Results and Discussion

### 3.1. Synthesis and Characterization on HPC-graft-AA (HPC-g-AA)

**Figure 1:**
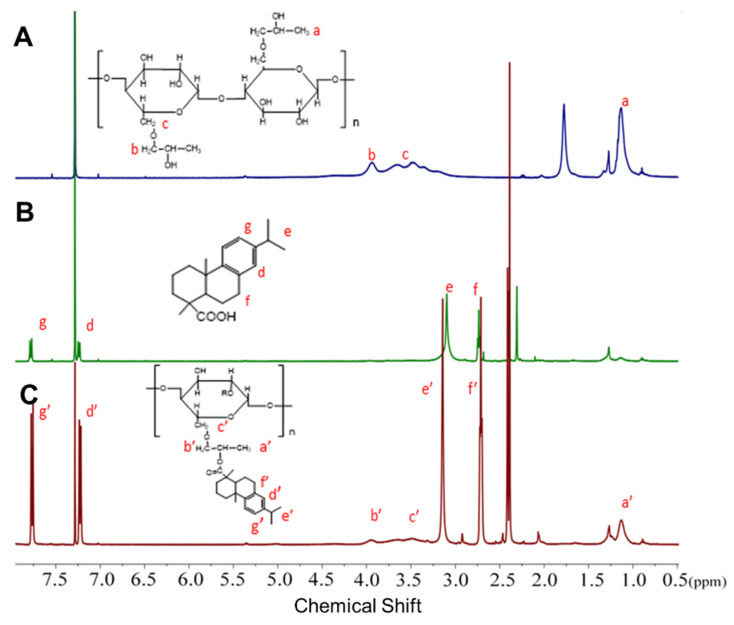
^1^H NMR spectra of (**A**) HPC; (**B**) abietic acid, and (**C**) HPC-*g*-AA.

To ensure injectability, the polymeric amphiphile with lower critical solution temperature (LCST) is probably a good choice because environmental temperature change would induce sol-to-gel phase transition and hydrogel formation. Herein, for constructing optimized HPC for the desired property of temperature-induced gelation, we coupled hydrophobic abietic moieties on the HPC chains (HPC-*g*-AA) to achieve its LCST approach to the human body temperature of 37 °C through acylation and esterification reactions in sequence (Scheme 1). The highest yield of the product was 82%, and stirring for a long amount of time did not decompose the product. The composition of the HPC-*g*-AA was evaluated by ^1^HNMR characterization. Figure 1 shows the typical ^1^HNMR spectra of HPC-*g*-AA. The proton locations were confirmed by comparison with spectra of the HPC and AA. The peaks at 7.2~7.8 ppm corresponded to aromatic protons from the abietic group on the pendant moiety (d′ and g′), along with the isopropyl proton from HPC (b′ and C′). A series of HPC-*g*-AA with different degrees of substitution (DS) could be prepared by editing the molar feed ratio of HPC/AA/Tos-Cl. These polymer characteristics are summarized in Table 2.

### 3.2. In Situ Gelatin and Thermoresponsive Behavior

**Figure 2:**
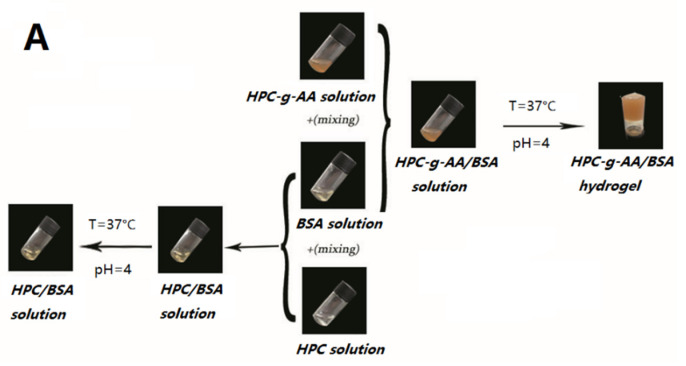

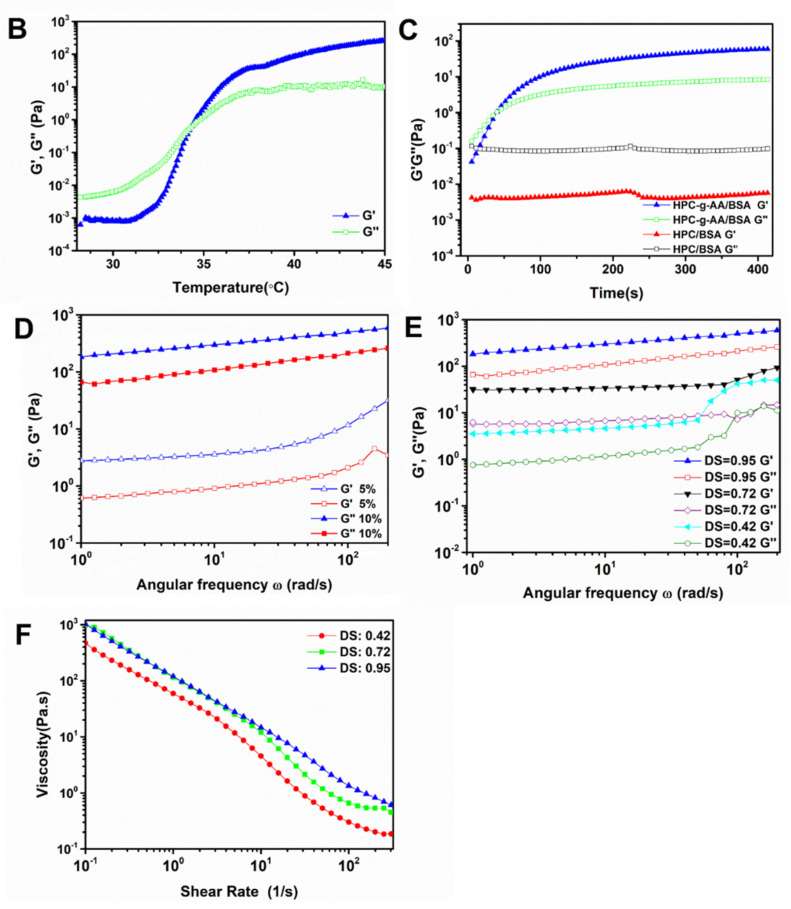
(**A**) Sol-to-gel phase transition photographs of HPC-*g*-AA/BSA hydrogels at 37 °C; (**B**) temperature sweep of HPC-*g*-AA/BSA hydrogels; (**C**) temperature sweep of HPC-*g*-AA/BSA hydrogels at 37 °C, a physical mixture of 10 wt% HPC-*g*-AA and 10% wt% BSA was used as a control; (**D**) G′ and G″ modulus of the HPC-*g*-AA/BSA hydrogel with concentration 5 wt% and 10 wt% from a frequency sweep; (**E**) G′ and G″ modulus of HPC-*g*-AA/BSA hydrogels with different DS; (**F**) G′ and G″ modulus and viscosity of the HPC-*g*-AA/BSA hydrogel from a frequency sweep.

To be injectable, a hydrogel should be liquid before and during the injection, but it should gelate after injection after a short amount of time, forming soft, self-supporting materials. In this work, the individual HPC-*g*-AA and BSA solutions (10 wt%) were both in sol state at a broad temperature region of 4−60 °C, as shown in Figure 2A; injectable, in situ-hardening hydrogels are rapidly created upon these two polymer dilute solutions being mixed in a pH = 4.0 solution at 37 °C. By contrast, no gelation phenomenon was observed when mixing the solutions of the HPC and BSA under the same conditions, or even the temperature greater than 60 °C. It should be noted that gel formation is not achieved when the pH of the mixed solution is higher than 4.0 due to the complete ionization of BSA at a pH higher than the isoelectric point (PI = 4.7) of BSA and consequent low hydrogen bonding interaction in the network. Therefore, in this work, the maximum pH of the BSA solution was set to 4.0.

We investigated the effects of temperature on the rheological properties of the hydrogel formulation by a continuous temperature sweep. The temperature was increased from 25 to 45 °C at a rate of 1 °C/min. The sol–gel phase transition temperature (T_gel_) is described as the temperature at which the storage modulus (G′) is the same as the loss modulus (G″). In Figure 2B, at temperatures below T_gel_, the HPC-*g*-AA/BSA system exhibits viscoelastic liquid behavior, and the curve shows the loss modulus dominant (G″ > G′). When the temperature increases at T > T_gel_, the storage modulus of the system comes to be superior (G′ > G″), suggesting that the solutions already turned into gels.

Rheological analysis of the storage modulus (G′) and loss modulus (G″) of the hydrogel formulations at 37 °C, was carried out to investigate the process of the gelation of HPC-*g*-AA/BSA hydrogels. Figure 2C shows the curve as a function of G′ and G″ vs. time at 37 °C for HPC-*g*-AA/BSA hydrogels: the G″ was above G′ in the initial stage, intimating that the hydrogel formulation mainly was the behavior of a low viscosity liquid; then the G′ increased significantly during 30–35 s as the result of the formation of the hydrogel networks; the gel point, defined as the crossover point of G′ and G″, was shown at around 35 s, much faster than that of the BSA gel at around 5–6 h at 60 °C, suggesting that our free-flowing polymer sol could transform into a non-flowing hydrogel while it was exposed to body temperature stimuli. The viscoelastic behavior of HPC-*g*-AA/BSA systems with different HPC-*g*-AA DS, HPC-*g*-AA concentrations, and BSA concentrations is summarized in Table 3. It was observed that gelling time was correlated with HPC DS, and less time was needed to form the gel when raising DS. When HPC was not coupled with hydrophobic moieties (AA), the gel point could not be observed, which agreed with the experimental results.

Additionally, when adjusting the ratio of HPC-*g*-AA to BSA while the total amount of polymer remained unchanged, the best gelation time and the storage modulus G′ of the HPC-*g*-AA/BSA hydrogel were inversely proportional to the concentration of BSA. The fastest gel time and best storage modulus appeared when the concentration of both was the same. The concentration of hydrogel formulation also dramatically influenced the G′ of the HPC-*g*-AA/BSA hydrogels, where a 1-fold rise in the concentration (5 to 10 wt%) resulted in 2~3-times higher G′ value. As shown in Figure 2D, 10 wt% of HPC-*g*-AA/BSA hydrogel had a storage modulus G′ of ~657Pa, nearly 2.6-fold of the 5 wt% gel (G′~253Pa).

G′ and G″ with frequency is called the “mechanical spectrum” of a hydrogel. The characteristic frequency sweep curve of HPC-*g*-AA/BSA hydrogels in Figure 2E has a solid-like character (G′) predominant over liquid-like, viscous response (G″), confirming its hydrogel behavior. It was found that the values of G′ were higher in the gel with higher DS, indicating that the mechanical properties of in situ-forming gels were closely related to the amphiphilicity of the HPC structure. Moreover, the HPC-*g*-AA/BSA hydrogel exhibited the physical nature of thixotropy. As shown in Figure 2F, the viscosity significantly decreasing with increasing shear rate at 37 °C suggests that the shear-thinning property of the hydrogels probably resulted from the disruption of physical crosslinking between the polymer chains with the shear stress. This shear-thinning characteristic could guarantee the injectability of the hydrogels and improve the delivery efficiency of drugs.

### 3.3. Formation Mechanism of HPC-g-AA/BSA Hydrogels

**Figure 3:**
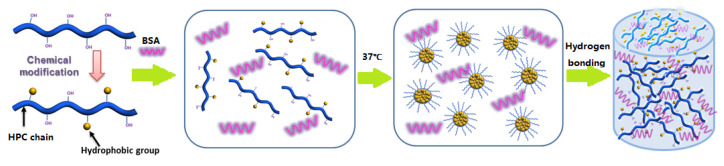
The scheme of the formation mechanism of HPC-*g*-AA/BSA hydrogels.

We suggest here a workable forming mechanism of the HPC-*g*-AA/BSA hydrogel. The in situ hydrogel-forming is due to the thermal gelation mechanism; hydrogen bonding interaction between the polymers promotes a physical crosslinking network and stabilizes and hardens the hydrogel. As shown in Figure 3, when the HPC-*g*-AA solution is exposed to 37 °C, the enrichment of hydrophobic interaction between HPC-*g*-AA chains triggers the phase separation via nucleation and growth mechanism. As phase separation proceeds, the dispersed droplets coarsen and increase the hydrogen bonding interaction between BSA and HPC-*g*-AA until the crosslinking network forms. The further increase in hydrogen bonding interaction before the gel point results in larger hydrogel modulus, also suggesting why the gelling time is proportional to the concentration of the formulation. Additionally, the HPC-*g*-AA/BSA hydrogel network formed by hydrogen bonds is not easy to break, so this hydrogel is thought to be thermally irreversible.

### 3.4. Cytotoxicity of DOX Delivered by Hydrogels

**Figure 4:**
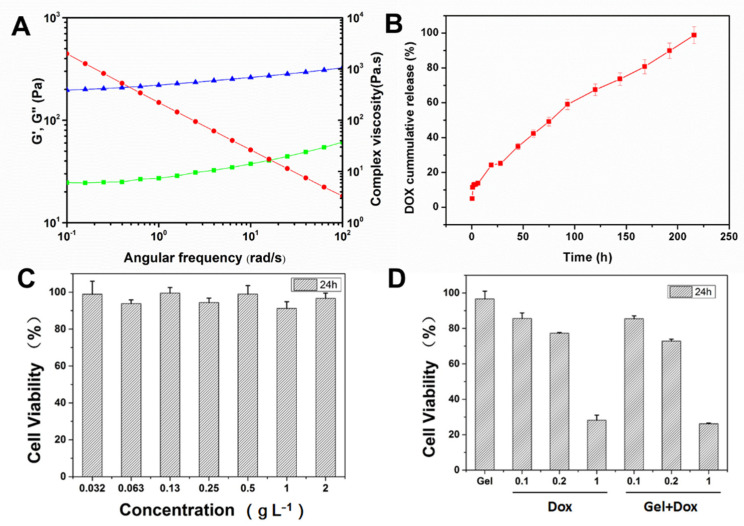
(**A**) G′ (blue)and G″ (green) modulus and viscosity (red) of DOX@HPC-*g*-AA/BSA hydrogel from frequency sweep; (**B**) the release kinetics curve (red) of DOX from DOX@ HPC-*g*-AA/BSA hydrogels; (**C**) in vitro cytotoxicity of HPC-*g*-AA/BSA hydrogel to 3T3; data are presented as the average ± standard deviation (n = 5); (**D**) cell viabilities of 4T1 cells after incubation with 5 μL of HPC-*g*-AA/BSA hydrogel only (Gel), free Dox (Dox), and DOX-loaded hydrogel (Gel + DOX). The DOX concentrations in the free Dox or group were 0.1 mg/mL, 0.2 mg/mL, and 1 mg/mL, respectively. (n = 3).

Low drug accumulation in tumors and significant side effects greatly limit an available chemotherapy drug’s clinical application and efficacy. The localized drug delivery system based on injectable hydrogel has tremendous advantages. For example, antitumor drugs could be continuously delivered to the tumor site in a targeted manner and effectively reduce systemic toxicity. The HPC-*g*-AA/BSA hydrogel has sol–gel transformation performance when responding to body temperature and good mechanical properties, rendering enormous potential for in vivo antitumor treatment, especially for intratumoral injection of chemotherapeutic agents, which could realize high local concentrations of the chemotherapeutics in the tumor. Thus, the behavior of local drug delivery of HPC-*g*-AA/BSA hydrogel was explored by incorporating a chemotherapeutic agent DOX into hydrogels (DOX@ HPC-*g*-AA/BSA hydrogel). We carried out rheology characterization to study the mechanical properties of the DOX@HPC-*g*-AA/BSA hydrogel. Frequency sweep studies witnessed that the loaded DOX reduced the hydrogel strength by about 2 times, as indicated by G′ and G″ (Figure 4A). The repulsive electrostatic interactions of DOX and BSA are the reason for the decrease in hydrogel strength. Next, we performed drug release kinetics studies of DOX from DOX@ HPC-*g*-AA/BSA hydrogels. The drug release curve (Figure 4B) revealed ~25% DOX release within 24 h from hydrogel, whereas the rest of the drug was released sustainedly for about 10 days.

In vitro biocompatibility of HPC-*g*-AA/BSA hydrogel was evaluated using MTT assay against 3T3 cells. The HPC-*g*-AA/BSA hydrogel exhibited significant non-toxicity with cell viability of about 97% up to 2 gL^−1^ (Figure 4C). Subsequently, the antitumor activity of DOX-loaded hydrogels with different DOX concentrations was investigated on 4T1 cells for 24 h. In Figure 4D, the cytotoxicity of DOX@ HPC-*g*-AA/BSA hydrogel against 4T1 cell line after 24 h incubation seems to have higher antitumor activity than free drug dose, verifying the advantage of DOX@ HPC-*g*-AA/BSA hydrogel as a drug carrier.

### 3.5. Subcutaneous Injectability and Antitumor Efficacy In Vivo

**Figure 5:**
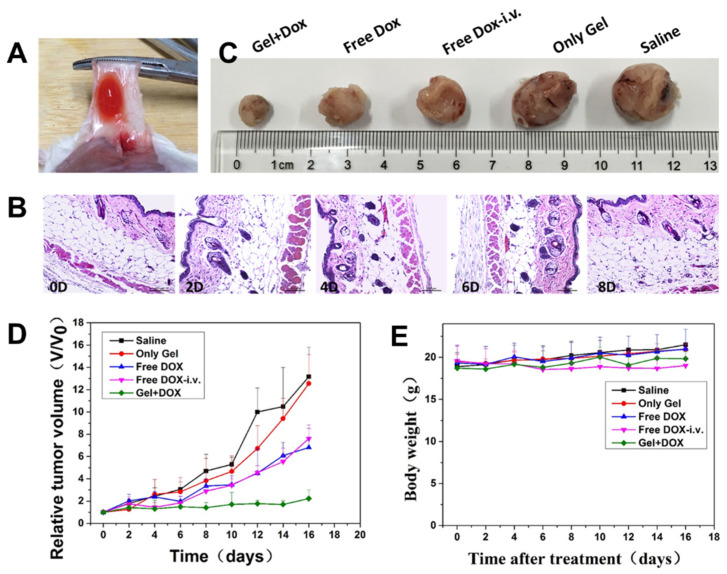
(**A**) In situ DOX@ HPC-*g*-AA/BSA formation in vivo; (**B**) the in vivo tissue biocompatibility of HPC-*g*-AA/BSA hydrogels at 10 min (0d), 2, 4, 6, and 8 days. The tissues surrounding the hydrogels were dissected and H&E stained; (**C**) the sizes of tumors obtained after the mice were sacrificed; (**D**) tumor growth inhibition with time; (**E**) the change in body weight of mice during treatments. In vivo antitumor effects of various treatments on BALB/c mice bearing 4T1. The groups were divided into saline, only gel, free DOX-i.v., free DOX, gel + DOX.

To investigate the subcutaneous injectability of the DOX@HPC-*g*-AA/BSA hydrogels, the hydrogel formulation was subcutaneously injected into the tumor sites of a mouse. After intratumoral injection, it should be noted that it is necessary to heat the tumor site to 37 °C to ensure gellation because the mouse’s body temperature is lower than that of human beings. About 10 min later, the DOX@ HPC-*g*-AA/BSA hydrogel was observed to create a red depot around the tumor when the skin was peeled off (Figure 5A). That is to say, compared with traditional chemotherapeutic agents, drugs and hydrogels are integrated into a structure in a short time, and a high local concentration of the drugs was achieved in target tumors, which prevented materials from flowing to other tissues and effectively inhibited the leaking of the drugs. Additionally, H&E images of the tissues surrounding the depot after intratumoral injection at 10 min (0d), 2, 4, 6, and 8 days, show no significant histological difference and no obvious inflammatory signs in Figure 5B. Therefore, the sol–gel phase transition properties of the HPC-*g*-AA/BSA hydrogel could allow minimal invasive administration into the deep sites of the tumors, which could minimize the discomfort and risk of infection.

**Figure 6:**
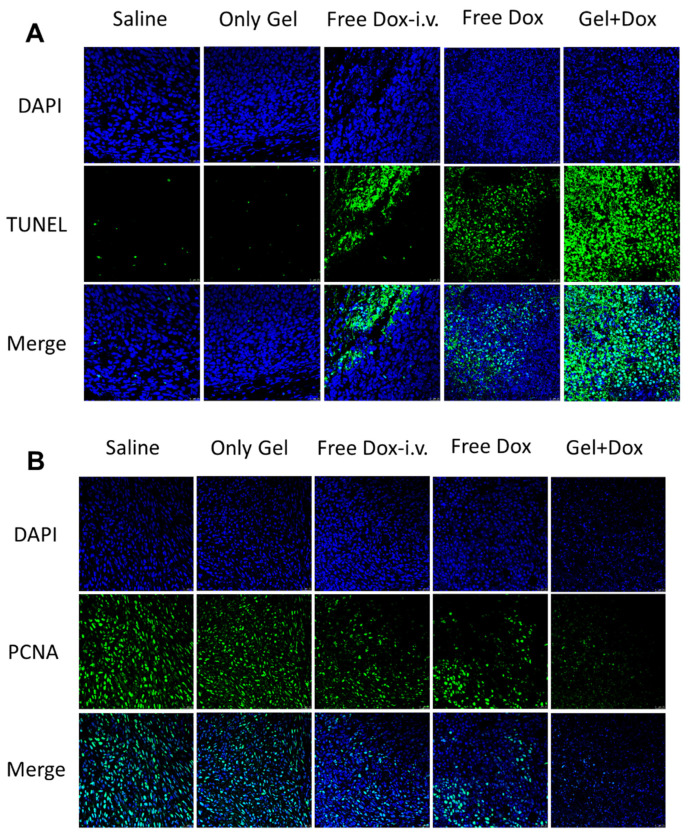
(**A**) Photographs of the TUNEL-stained fluorescent microscopy of different tumor slices; (**B**) photographs of the PCNA-stained fluorescent microscopy of different tumor slices.

Next, the therapeutic efficacy of drug-loaded hydrogels in vivo was studied. DOX@ HPC-*g*-AA/BSA hydrogels injected subcutaneously (s.c.) were compared with both i.v. and s.c. administration of DOX solution in the 4T1 tumor-bearing mouse model. The mice were divided into five groups consisting of control injected with physiological saline (s.c.); DOX in solution (i.v.); DOX in solution (s.c.); blank gels, and DOX-loaded gels (s.c.). The same tumor growth trend was observed in the blank gel-treated group and the saline group, of which the sizes of the tumors increased speedily in 16 days (Figure 5C), indicating that the hydrogel has no cytotoxic effect on tumor cells. It was noted that at the time of dissection, there was no hydrogel residue presenting on the treated site, indicating that the hydrogel had been degraded completely. On the contrary, tumor growth was inhibited at different degrees for all drug treatment groups. The DOX-loaded gels treated group had the strongest inhibitory effect on tumor growth, and tumor inhibition rate (TIR) was the highest (93.1%) after 16 days, which was more evident than that of DOX solution (both i.v. and s.c.) (Figure 5D). The hydrogel localized DOX at the tumor site at higher levels over a more extended period and enabled DOX to exert a cytotoxic effect against the cancer cells. Photographs of the PCNA-stained and TUNEL-stained fluorescent microscopy of tumor slices again confirmed that DOX@ HPC-*g*-AA/BSA hydrogels induced the strongest apoptosis in tumor cells than the other treatments (Figure 6A,B), further illustrating the higher antitumor efficacy of delivery of the HPC-*g*-AA/BSA hydrogel. Moreover, since no weight loss was observed in any mice during the treatment, the therapy was well tolerated (Figure 5E). The significantly improved antitumor effectiveness was probably due to high drug accumulation at the tumor site and the long retention time of hydrogels. Therefore, all results confirm the advantage of the DOX@HPC-*g*-AA/BSA hydrogels for localized antitumor therapy.

### 3.6. Histological Examination

**Figure 7:**
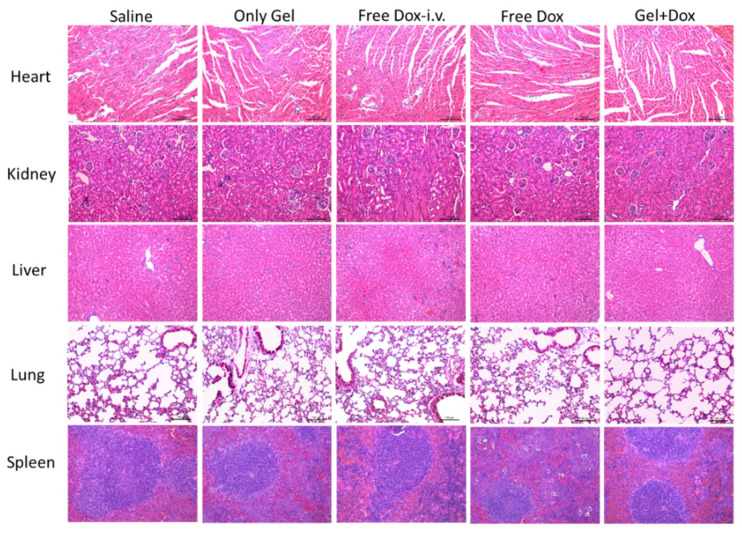
Histological H&E staining analyses of the organs of mice after different treatments. Nuclei were stained blue, and extracellular matrix and cytoplasm were stained red.

Finally, the biocompatibility of HPC-*g*-AA/BSA hydrogel was primarily evaluated by systemic toxicity. As shown in Figure 7, the DOX-loaded gels group showed relatively typical histological morphology, and no damage was observed in H&E stained slices of heart, liver, spleen, lung, and kidney, demonstrating good biological safety of HPC-*g*-AA/BSA hydrogels. The decrease in systemic toxicity of drug-loaded hydrogels should be attributed to the local and delayed release at the tumor site, indicating that the DOX-incorporated HPC-*g*-AA/BSA hydrogels exhibit good safety and biocompatibility.

## 4. Conclusions

We constructed in this work an in situ-forming injectable and cellulose/albumin-based hydrogel that responds to the change in physiological temperature by hydrophobic and hydrogen bonding interactions. The hydrogels formed from biomaterials HPC-*g*-AA and BSA exhibited higher mechanical strength, temperature response, and sustained release. The HPC-*g*-AA/BSA injectable hydrogel overcame needle blockage and made the hydrogel reach the deep implant tissue easily, so it has more advantages for localized cancer therapy application. In vitro/in vivo biocompatibility investigation disclosed the non-cytotoxic nature of the HPC-*g*-AA/BSA hydrogels. Sustained delivery of DOX from the gel implanted in the vicinity of the tumor resulted in an increased anticancer effect.

In summary, the thermosensitive nature and prolonged drug release make HPC-*g*-AA/BSA injectable hydrogel promising material for localized drug delivery. Moreover, this hydrogel probably has bright prospects in regenerative medicine, tissue engineering, stimuli-responsive 3D cell culturing, etc.

## Figures and Tables

**Table 1:** Experimental set up of the rheological characterization.

Experiment	Measured Parameters	Temperature (°C)	Strain (%)	Frequency (Hz)
Time sweep	G′, G″, gelling time	37	0.1	0.16
Frequency sweep	G′, G″	37	0.1	0.16–31.83
Temperature sweep	G′, G″, T_gel_	25–45	0.1	0.16

**Table 2:** HPC-*g*-AA characteristics with DS.

	Molar Feed Ratio (HPC_unit_/AA/Tos-Cl)	DS	Yield (%)	LCST (°C)
HPC-*g*-AA-1	1:0.6:0.6	0.42	76.2	39
HPC-*g*-AA-2	1:0.8:0.8	0.72	62.1	37
HPC-*g*-AA-3	1:1:1	0.95	81.7	35

**Table 3:** The sol/gel transition temperature and gelling time at 37 °C of HPC-*g*-AA/BSA hydrogel as a function of HPC-*g*-AA DS, HPC-*g*-AA concentration, and BSA concentration.

HPC-*g*-AA		BSA	HPC-*g*-AA/BSA Hydrogel		
DS	Concentration (wt%)	Concentration (wt%)	T_gel_ (°C)	Gelling Time (s)	G′ (Pa)
0.95	3.0	7.0	36	410	264
0.95	5.0	5.0	35	40	632
0.95	7.0	3.0	34	180	97
0.72	3.0	7.0	37	312	276
0.72	5.0	5.0	37	212	233
0.72	7.0	3.0	36	198	115
0.42	3.0	7.0	37	804	168
0.42	5.0	5.0	37	412	210
0.42	7.0	3.0	37	500	65
0	5.0	5.0	-	-	-

## Data Availability

The data presented in this study are available on request from the corresponding author.
